
JOURNAL INFORMATION
==============================
NLM Title Abbreviation: Innov Surg Sci
Journal ID: Innov Surg Sci
NLM Journal ID: 101708165
Journal ID: iss

Innovative Surgical Sciences

EISSN: 2364-7485
Publisher: De Gruyter

ARTICLE INFORMATION
==============================
PMCID: PMC10216887
DOI: 10.1515/iss-2023-9002
Article ID: iss-2023-9002
Article version: 1
Subjects: Article

Abstract DGPRÄC

Publication date: 2023 Mar
Electronic publication date: 2023 Mar 13
Volume: 8
Issue: Suppl1
First page: 10
Last page: 18
Copyright: © the Author(s), published by De Gruyter
Copyright year: 2023
Copyright holder: the Author(s), published by De Gruyter
License: This work is licensed under the Creative Commons Attribution 4.0 International License.
License URL: https://creativecommons.org/licenses/by/4.0/

Page count: 9

==============================

### Adipose tissue derived stem cells from affected and unaffected tissues can be used to study pathogenesis of rare disease Multiple Symmetric Lipomatosis

ID: 66

**Kategorie: DGPRÄC - Forschung in der Plastischen Chirurgie**

Lukas Prantl[1], Oliver Felthaus[2], Andreas Eigenberger[2]

[1]*Universität Regensburg, Hochschulzentrum für Plastische, Ästhetische, Hand- und Wiederherstellungschirurgie, 93053 Regensburg, Deutschland*, [2]*Universität Regensburg, Hochschulzentrum für Plastische Chirurgie, Applied Stem Cell Research Center, Regensburg, Deutschland*

**Background:**

Multiple symmetric lipomatosis is a rare disease characterized by the excessive growth of uncapsulated masses of adipose tissue. Although the etiology has yet to be elucidated, a connection to brown adipose tissue has been proposed recently. The mTOR pathway was found to be regulated in lipomatous tissue as well as associated with brown adipose tissue.

**Materials and methods:**

Affected and unaffected tissues were compared morphologically. Adipose-derived stem cell cultures from the tissue samples were generated and characterized as a possible cellular model of multiple symmetric lipomatosis. The proliferative activity, differentiation potential, and gene expression were evaluated. Additionally, the cells were treated with mTOR inhibitors. Secretome of cells from affected and unaffected areas was evaluated using mass spectrometry.

**Results:**

Stem cells from lipomatous tissue showed significantly higher proliferative activity. Polymerase chain reaction arrays showed marked changes in genes associated with proliferation, hormonal regulation, and mitochondria. Multiple symmetric lipomatosis tissue is morphologically and histologically different from regular subcutaneous fat. The differences in both proliferation and differentiation between adipose tissue derived stem cells (ASCs) from lipomatous and normal tissue decreased after mTOR pathway inhibition. Mass spectrometry revealed significant differences in secreted proteins.

**Conclusion:**

This study indicates an involvement of mesenchymal stem cells in the pathogenesis of multiple symmetric lipomatosis Adipose-derived stem cells exhibit many characteristics previously described for native multiple symmetric lipomatosis fat tissue and propose that they are therefore an excellent tool for further functional investigations in multiple symmetric lipomatosis and other disorders of the fat tissue. Treatment with mTOR inhibitors might be a novel therapeutical approach for patients suffering from multiple symmetric lipomatosis

Open Access. © 2023 The Author(s), published by De Gruyter. This work is licensed under the Creative Commons Attribution 4.0 International License.

### Functional Outcome of Dual Reinnervation with Cross-Facial Nerve Graft and Masseteric Nerve Transfer for Facial Paralysis

ID: 141

**Kategorie: DGPRÄC - Forschung in der Plastischen Chirurgie**

Branislav Kollár[1], Jakob Weiss[1], Jurij Kiefer[1], Steffen Ulrich Eisenhardt[1]

[1]*Universitätsklinikum Freiburg, Klinik für Plastische und Handchirurgie, Freiburg im Breisgau, Deutschland*

**Background:**

The combination of Cross-Facial Nerve Graft (CFNG) and Masseteric Nerve Transfer (MNT) for reinnervation of facial paralysis may provide advantages of both neural sources. However, quantitative functional outcome reports with a larger number of patients are lacking in the literature. Here we provide our 8-year experience with this operative technique.

**Materials and methods:**

Twenty patients with complete facial paralysis (duration of paralysis 8,45±3,63 months) received dual reinnervation with CFNG and MNT. The functional outcome of the procedure was evaluated with the physician-graded outcome metric eFACE. The objective artificial intelligence-driven softwares Emotrics and FaceReader were used for oral commissure measurements and emotional expression assessment, respectively.

**Results:**

The mean follow-up was 31.75±23.32 months. In the eFACE score, the nasolabial fold depth and oral commissure at rest improved significantly ($p<0.05$) towards a more balanced state after the surgery. Postoperatively, there was a significant decrease in oral commissure asymmetry while smiling (19,22±6,1mm to 12,19±7,52mm). For the emotionality expression, the median intensity score of happiness, as measured by the FaceReader software, increased significantly while smiling (0.28, IQR 0.13-0.64). Seven (35%) patients reported a spontaneous smile. In 5 (25%) patients, a secondary static suspension of the mouth with fascia lata strip had to be performed due to an unsatisfactory resting symmetry.

**Conclusion:**

The MNT provided reliable voluntary motion and the CFNG could contribute to good resting tone in the majority of the patients, thus merging the advantages of both neural sources without additional morbidity. Despite the early reinnervation, spontaneity is less predictable to achieve.

**The Value of Negative-Pressure Wound Therapy and Flap Surgery in Hidradenitis Suppurativa – A Single Center Analysis of Different Treatment Options**

ID: 166

**Kategorie: DGPRÄC - Forschung in der Plastischen Chirurgie**

Maximilian Stumpfe[1], Ingo Ludolph[1], Andreas Arkudas[1], Aijia Cai[1], Wibke Müller-Seubert[1], Theresa Promny[1], Raymund E. Horch[1]

[1]*Universitätsklinikum Erlangen, Plastisch- und Handchirurgische Klinik Universitätsklinikum Erlangen, Erlangen, Deutschland*

**Background:**

Hidradenitis suppurativa is manifested by painful abscesses and scarring of sweat glands. Axillary, inguinal and genital regions are mostly affected. Multiple options exist in the treatment of hidradenitis suppurativa. The aim of this retrospective, mono-center cohort study was to analyze the outcome of different treatment methods after radical excision of hidradenitis suppurativa.

**Materials and methods:**

We retrospectively evaluated the treatment strategy and recurrence rate of hidradenitis suppurativa. We included all eligible patients of legal age between February 2003 and October 2021, with the diagnosis of Hidradenitis suppurativa and the necessity for surgical treatment. All patients with surgical treatment and direct wound closure by suture were excluded. Bacterial load and flora were analyzed for primary and secondary reconstruction in combination with negative-pressure wound therapy. Patient data were analyzed for recurrence rate and remission time according to different reconstructive techniques.

**Results:**

In 44 affected anatomical sites (n = 23 patients) we treated 15 patients with negative-pressure wound therapy. Bacterial load and flora were lower in the last wound swab of patients with multi-surgical procedures (22 localizations) compared to the first wound swab independent of the use of negative-pressure wound therapy. Wound closure, independent of a direct and multi-stage procedure was achieved by local fasciocutaneous flaps (n = 12), secondary intention healing (n = 7), secondary intention healing with buried chip skin grafts (n = 10), or split-thickness skin grafts (n = 15). Radical excision combined with split-thickness skin grafts showed the lowest recurrence rate in the follow-up (16%; n = 4).

**Conclusion:**

Radical excision of hidradenitis suppurativa as gold standard for surgical treatment combined with negative-pressure wound therapy as multi-stage procedures ultimately reduced bacterial load and flora in our study. The use of split-thickness skin grafts showed the lowest recurrence rate.

**Perioperative Use of Vasopressors in Free Flap Transplants: A Retrospective Study**

ID: 296

**Kategorie: DGPRÄC - Forschung in der Plastischen Chirurgie**

Nadjib Dastagir[1], Doha Obed[1], Khaled Dastagir[1], Peter M. Vogt[1]

[1]*Hannover, Medizinische Hochschule Hannover, Hannover, Deutschland*

**Background:**

The perioperative use of vasopressors in free flap surgeries is controversially debated. The predominant concern is pedicle blood supply will be negatively affected, thus leading to increased post-operative complications. However, little is known about the relationship between vasopressor use and its impact on intrinsic blood supply of free flaps. Comorbidities that diminish blood circulation may cause a higher risk of necrosis from vasopressor use due to the decreased intrinsic free flap blood supply. The aim of this study is to establish the role of peri-operative vasopressors in free flap necrosis based on flap localization and patients' comorbidities.

**Materials and methods:**

We retrospectively analyzed 106 patients that received free flap treatment between 2006 and 2020 stratifying based on age, sex, body mass index (BMI), and smokers vs non-smokers. We assessed the role of mean arterial pressure (MAP) and the perioperative use of catecholamine during free flap surgeries using univariate and multivariate analyses.

**Results:**

The use of fasciocutaneous flaps, especially on breast and extremities in patients with vascular disease, had the highest risk for marginal flap necrosis (OR: 1.64, p=0.01). Musculocutaneus free flaptransplanted on central body defects were less affected by catecholamine use (OR: 1.01 p=0.05). In summary fasciocutaneous flaps used on extremities in patients with cardiovascular disease showed the greatest vulnerability to high vasopressor use. Additionally, we identified that low MAP (<65mmHg) in patients with peripheral artery disease (PAD) led to increased flap marginal necrosis (OR: 1.32 p=0.02).

**Conclusion:**

To minimize the risk of flap marginal necrosis in patients with cardiovascular disease, we recommend limited use of catecholamine or minimizing the flap size, particularly when using fasciocutaneous flaps to cover defects on extremities. Maintaining a mean arterial pressure above 65mmHg in patients with PAD was beneficial.

**The role of Nature Killer (NK) Cells in P3 digit tip regeneration**
ID: 299

**Kategorie: DGPRÄC - Forschung in der Plastischen Chirurgie**

Nadjib Dastagir[1], Doha Obed[1], Khaled Dastagir[1], Peter M. Vogt[1]

[1]*Hannover, Medizinische Hochschule Hannover, Hannover, Deutschland*

**Background:**
Limb regeneration in mammals has been shown to be less comprehensive compared to amphibian models. Part of the reason for this difference in regenerative capacity is due to the immune system. The effects of immune cells, such as macrophages, on regeneration have been well examined, however the role of Nature Killer (NK) cells remains largely unknown. Here, we demonstrate the importance of NK cells in regeneration and that the effects of these cells depend on tissue origin.

**Materials and methods:**
Amputation of the distal one-third of the terminal phalanges was performed on aged matched 8-10 week old immunodeficient NSG mice. Two digits per hind paw (digits 2 and 4) were amputated. Digit number 3 functioned as an uninjured control. Adoptive cell transfer (ACT) of flow cytometry purified splenic NK (SpNK) or thymic NK (ThNK) cells from C57BL/6 mice were injected via the tail vein. Changes in hard and soft tissue volume were assessed using microCT and histology. To determine immune cell presence and receptor expression in the regenerating digit tip we used immunofluorescent staining and flow cytometry.

**Results:**
We confirmed NK cell recruitment to the regenerating digit tip. NK cell cytotoxicity was observed against osteoclast and osteoblast progenitors. ACT of ThNK cells induced apoptosis with a reduction of osteoclasts, osteoblasts, and proliferative cells, resulting in inhibition of regeneration. By contrast, ACT of splenic NK cells showed reduced cytotoxicity towards progenitor cells and improved regeneration. Adoptive transfer of NK cells deficient in NK cell activation genes identified that promotion of regeneration by SpNK cells requires Ncr1, whereas inhibition by ThNK cells is mediated via Klrk1 and perforin.

**Conclusion:**
These findings yield insight into mammalian digit tip regeneration and demonstrate the importance of NK cells on regenerative ability. Successful future therapies aimed at enhancing regeneration will require a deeper understanding of progenitor cell protection from NK cell cytotoxicity.

**Zuverlässigkeit und onkologische Sicherheit der mikrochirurgischen Rekonstruktion knochen-assoziierter Sarkome in der interdisziplinären Plastischen Chirurgie - Efficacy and oncological safety of microsurgical reconstruction in the interdisciplinary treatment of sarcoma affecting the bone**

ID: 833

**Kategorie: DGPRÄC - Interdisziplinäre Zusammenarbeit zur Verbesserung der Versorgungsqualität – Fallbeispiele**

Jurij Kiefer[1], Johannes Zeller[1], Daniel Kraus[1], Steffen Ulrich Eisenhardt[1]

[1]*Universitätsklinikum Freiburg, Klinik für Plastische und Handchirurgie, Freiburg, Deutschland*

**Background:**

Sarcomas are a rare and highly heterogeneous group of malignancies. Sarcomas affecting the bone require an interdisciplinary approach to resection and reconstruction. However, microsurgical reconstruction strategies must not negatively impact oncological safety and survival. Here, we analyzed the oncosurgical safety, including survival and overall complication rates in patients with bone-associated sarcomas that underwent complex microsurgical procedures for limb salvage.

**Materials and methods:**

We performed a retrospective chart review of all patients treated for soft tissue and bone sarcoma at our institution, focusing on bone affection and microsurgical reconstruction between 2000 and 2019. This subgroup was further investigated for tumor resection status, 5-year survival rate, length of hospital stay, and overall complication and amputation rates.

**Results:**

Between 2000 and 2019, 803 patients underwent sarcoma resection and reconstruction in the Department of Plastic and Hand Surgery. Of these, 212 patients presented with sarcoma of the extremity affecting the bone. Within this subgroup, 40 patients required microsurgical reconstruction for limb salvage, which was possible in 38 cases. R0 resection was achieved in 93.8 %. The 5-year survival was 96.7 %, and the overall complication rate was 25 %, of which 40 % were microsurgery-associated complications.

**Conclusion:**

Safe and function-preserving treatment of bone-associated sarcoma is challenging. Primary reconstruction with microsurgical techniques of sarcoma-related defects enables limb-sparing and adequate oncosurgical cancer treatment without increasing the risk for local recurrence or prolonged hospital stay.

**Fluid and Burns: what we know and what we don't know – retrospective analysis of 2576 patients (children and adults)**

ID: 497

**Kategorie: DGPRÄC - Verbrennungen bei Kindern**

Vasileios Vasileiadis[1], Annette Aigner[2], Konrad Reinshagen[3], Ingo Koenigs[1]

[1]*Altonaer Kinderkrankenhaus, Hamburg, Deutschland*, [2]*Charité – Universitätsmedizin Berlin, Institute of Biometry and Clinical Epidemiology, Berlin, Deutschland*, [3]*Universitätsklinikum Hamburg-Eppendorf, Pediatric Surgery, Hamburg, Deutschland*

**Background:**

Fluid resuscitation is of great importance in the management of burns. Various formulae have been described for calculating fluid management especially in severely burned patients. Although the parkland formula is widely used, its effectiveness and clinical value are discussed controversial. The impact of calculated volume of fluid by Parkland on the outcome of burned patients was investigated.

**Materials and methods:**

All patients with thermal injuries of the German burn registry (VR-DGV-project-ID 2020/01) between January 2016 and December 2020 with an affected body surface area more than 15% were analysed. Patients were divided into two groups according to their age: paediatric and adult. Outcome was compared in correlation with the volume of fluid given according to Parkland formula and analysis was performed with logistic regression.

**Results:**

480 children (0-15 years old) and 2096 adults (16-100 years old) were included in the analysis. In the paediatric group the logistic regression analysis showed a low correlation between Parkland formula and given volume 0.78 (95% CI 0.72-0.83). Apparently, all centres' fluid management deviated from calculation of parkland formula with the majority over-resuscitating regardless the size of the centre. The deviation seemed to depend on the cause of accident. Less given volume than calculated with Parkland formula reduced the length of hospital stay. Only 5 children died, all received more fluid than Parkland's estimation. In the adult group, a negative deviation from parkland seemed to correlate with shorter length of hospital stay, whereas it had no influence on mortality.

**Conclusion:**

This retrospective analysis provided no clear evidence of the eligibility of the parkland formula. Even huge data sets of a burn registry such those of the German Society for Burn Medicine are limited in answering the "fluid question" definitively. Extended parameters such as urine output and blood pressure would be of great interest. A randomized study with low – medium – high fluid income groups would be preferable but would most likely provoke ethical questions and ambiguous acceptance in clinical practice.
